# Major challenges in youth psychopathology: treatment-resistant depression. A narrative review

**DOI:** 10.3389/fpsyt.2024.1417977

**Published:** 2024-07-11

**Authors:** Giulia Menculini, Gianmarco Cinesi, Francesca Scopetta, Matteo Cardelli, Guido Caramanico, Pierfrancesco Maria Balducci, Filippo De Giorgi, Patrizia Moretti, Alfonso Tortorella

**Affiliations:** ^1^ Section of Psychiatry, Department of Medicine and Surgery, University of Perugia, Perugia, Italy; ^2^ Community Mental Health Center “CSM Terni”, Department of Psychiatry, Local Health Unit USL Umbria 2, Terni, Italy; ^3^ Division of Psychiatry, Clinical Psychology and Rehabilitation, General Hospital of Perugia, Perugia, Italy

**Keywords:** major depressive disorder, treatment-resistant depression, adolescents, youth psychopathology, fast-acting antidepressants, ketamine, esketamine, glutamate

## Abstract

Major depressive disorder (MDD) represents a major health issue in adolescents and young adults, leading to high levels of disability and profoundly impacting overall functioning. The clinical presentation of MDD in this vulnerable age group may slightly differ from what can be observed in adult populations, and psychopharmacological strategies do not always lead to optimal response. Resistance to antidepressant treatment has a prevalence estimated around 40% in youths suffering from MDD and is associated with higher comorbidity rates and suicidality. Several factors, encompassing biological, environmental, and clinical features, may contribute to the emergence of treatment-resistant depression (TRD) in adolescents and young adults. Furthermore, TRD may underpin the presence of an unrecognized bipolar diathesis, increasing the overall complexity of the clinical picture and posing major differential diagnosis challenges in the clinical practice. After summarizing current evidence on epidemiological and clinical correlates of TRD in adolescents and young adults, the present review also provides an overview of possible treatment strategies, including novel fast-acting antidepressants. Despite these pharmacological agents are promising in this population, their usage is expected to rely on risk-benefit ratio and to be considered in the context of integrated models of care.

## 1 Introduction: major depressive disorder in youth populations

Major depressive disorder (MDD) is a serious psychiatric disorder with a relevant impact on quality of life and overall functioning. According to the World Health Organization (WHO), MDD represents the main cause of years lived with disability worldwide, leading to decreased involvement in social and work activities and to increased medical comorbidities and health resource use (1). The lifetime prevalence of MDD may reach up to 30% in special populations (2), and is usually higher among women (3).

Depressive disorders, and particularly MDD, also represent a relevant health issue in adolescents and young adults, with an overall prevalence estimated around 2-3% (4, 5), reaching up to 20% at the end of puberty (6). The incidence of MDD during adolescence is estimated about 7.5% (2.3% for serious forms of the disorder) (7), with higher prevalence rates among young women (8). A meta-analysis of 80,879 youths conducted during the first year of the Coronavirus disease 2019 (COVID-19) pandemic concluded that the global prevalence of youths experiencing clinically significant depressive symptoms increased to 25% (9). Adolescence is a vulnerable period for developing mental health issues, and particularly depression, due to the interaction of different factors encompassing biological, environmental, and social determinants (10). Indeed, the onset of puberty, together with the exposure to social media, bullying and cyber-bullying episodes and education-related issues, make this life period extremely prone to the onset of psychopathology (11). Adolescents and young adults experience a number of symptoms during depressive episodes, including persistent sadness, irritability, weight change, loss of energy, and insomnia (12). The emergence of MDD during adolescence is associated with significant functional impairment and higher comorbidity rates (11), including medical diseases (13), as well as with an increase in substance abuse (14). More than 30% of youths suffering from MDD experience suicidal ideation, and over 10% attempt suicide, the latter being the second cause of death among youths aged 15-24 (15). Studies conducted among university students highlighted that MDD could seriously impact academic performances and lead to impaired social relationships and low self-esteem (16). Moreover, the onset of MDD during youth may lead to higher recurrence and relapse rates during the following years (17). Despite the relevance of MDD among youths under a clinical and epidemiological point of view, young people suffering from this disorder are often not likely to seek help. Hence, according to recent reports, only 35% of adolescents suffering from this condition accessed mental health resources and only 33% received adequate treatment (8).

In this narrative review, we decided to focus on one of the major challenges posed by MDD, which is treatment-resistance. Since a considerable percentage of MDD first episodes occur during adolescence or young adulthood, we believe that the appropriate identification of difficult-to-treat conditions is crucial to prevent functional impairment and chronicization during the following years. As a consequence, the main aim of this paper is to critically summarize evidence concerning treatment-resistant depression (TRD) in youths, with specific focus on: definition, epidemiology, impact, clinical correlates (including differential diagnosis issues), and possible treatment strategies, with particular interest in novel antidepressant strategies. To this attempt, we performed a literature review, variously combining the following keywords in PubMed, Scopus, and Web of Science databases: “major depressive disorder”, “depressive disorders”, “treatment-resistant depression”, “treatment resist*”, “youth*”, “adolescent*”, “young adult*”.

## 2 Treatment-resistant depression in youth populations: clinical challenges and impact

The currently accepted definition of TRD refers to a condition in which subjects do not respond, or reach remission, after treatment with at least two antidepressants at adequate dose and for an adequate period of time (18). Using this definition, the prevalence of TRD is estimated about 20-30% among subjects suffering from MDD (19), but rates vary from 12% to 55% (20). This huge variability is mainly due to the lack of homogenous criteria for TRD, as well as to various staging models that consider different number of failed antidepressant trials and different possible treatments, e.g., variably including also psychotherapies and electro-convulsant therapy (ECT) (21). Despite consensus has not been reached yet, most studies consider response as a reduction in depressive symptoms of at least 50%, as evaluated by well-validated rating scales (22). The minimum duration of antidepressant treatment should be 4 weeks (23), with a variable range of 4-12 weeks (24). Overall, subjects with MDD who undergo adequate treatment usually reach remission in 30% cases. Out of the remaining 70%, about 20% respond to treatment without reaching remission, while 50% do not respond at all (25). To note, the possibility for reaching remission significantly decreases after the second and third treatment strategy, as detected in the wide multicenter Sequenced Treatment Alternatives to Relieve Depression (STAR*D) study (26). Furthermore, in a relevant percentage of cases where a response is observed, residual symptoms may be present and impact overall quality of life of affected individuals (27).

TRD represents a complex clinical entity, underpinning different depression subtypes, as well as psychiatric and medical comorbidities (28). This condition should be conceptualized as a phenomenon that lies on a continuum ranging from partially responsive depression to multi-treatment resistant MDD (29). As we will elucidate later, TRD is also a multifaceted phenomenon, since several factors lead to reduced treatment effectiveness in MDD. Among possible risk factors for TRD, psychiatric comorbidities, particularly anxiety disorders (30, 31), psychotic features (18), and poor treatment adherence (32) have pointed out as the most common ones.

In youth populations, no specific criteria for TRD have been suggested, and research on the topic is scant (33, 34). A broad definition proposed for TRD in adolescents is a depressive disorder that does not respond to a two-month antidepressant treatment, namely a drug prescribed at a dose equivalent to 40 mg of fluoxetine and/or 8-16 sessions of cognitive-behavioral or interpersonal therapy (35, 36). Psychopharmacological treatment efficacy should be evaluated at intervals of at least four weeks, increasing doses in case of incomplete response (37). The main strength of this definition is the inclusion of psychotherapy among possible treatment strategies, which lacks in most adult TRD definitions (21). Indeed, psychotherapy demonstrated its efficacy in youth depression, alone or associated with pharmacological interventions, and is mentioned in the majority of treatment guidelines for this population (37–40). On the other hand, the different response to antidepressants in adolescents is not taken into account and the doses are similar to those advised for adults. It has anyway been largely demonstrated that young populations can develop activation symptoms, mood lability, and irritability in response to conventional antidepressant treatments, possibly leading to worsening of depression and suicidality (35). This is a major issue that experts should consider when defining TRD in adolescent and young adults, possibly reaching a more population-specific and comprehensive definition.

Based on the currently accepted description, a consistent percentage of adolescents and young adults with MDD – estimated around 40% - fail to respond to treatment with an antidepressant medication or evidence-based psychotherapy (33, 41), resulting in what is commonly referred to as TRD (34). To note, there is also a proportion of patients – about one third – initially reaching remission who do not maintain this outcome in the long-term (42). The impact of TRD on overall well-being has widely been recognized (43), together with the significant increase in mortality risk among affected individuals (44, 45). These aspects are particularly meaningful in youth populations. Indeed, TRD is associated with cognitive impairment (46), reduced coping abilities (47), and higher risk of developing medical diseases (48, 49), resulting in greater overall severity, poorer outcomes, and reduced functioning in different areas (50–52). High mortality rates are linked to comorbidities and to increased suicide risk (53). In most cases, TRD leads to higher use of healthcare resources, with subsequent increased health costs (54–56), which is even more relevant if we focus on young populations at their very first working experiences. As a result, quality of life is significantly impaired in adolescents and young adults suffering from TRD (57).

## 3 Clinical correlates of TRD in youth populations

The clinical presentation of MDD in youths, and particularly adolescents, can significantly differ from what can be observed in adult populations, although diagnostic criteria are the same. Depressive symptoms can sensitively be different across the lifespan, which lead to low diagnostic validity of traditional nosographic categories in this population (58). In early and mid-adolescents some features, particularly irritability, somatic symptoms, and anxiety, can be ever more prevalent than low mood and sadness, while in older adolescents and young adults affective and cognitive symptoms are prominent and closely resemble those observed in adults (59). Among youths, somatic and autonomic symptoms - including eating and sleep disturbances – could in some cases prevail on cognitive features and anhedonia and may lead to increased duration and severity of the depressive episode (36). Moreover, different clinical pictures may be observed also based on possible pathways leading to the development of depressive symptoms and depressive disorders. The impact that substance abuse, as well as the co-occurrence of behavioral addictions, e.g., pathological internet and social media use, can exert on the clinical picture may indeed be crucial. Sex differences have also been described, including a higher prevalence of eating and body image disorders in females, while somatic symptoms, attention deficits, restlessness, and anhedonia are more frequent in males, increasing the risk of developing conduct disorders and substance use (59). Another not negligible influence is represented by cultural and societal determinants, since the development of depression may be affected by specific factors, e.g., belonging to a minority or being culturally vulnerable in challenging environments, such as huge urban contexts (60, 61). This is a crucial points if we consider the progressive increase of migrant families belonging to ethnic minorities in European countries and the high prevalence of mood disorders in this population (62), with major challenges for psychiatric care. Indeed, symptom presentation significantly vary across cultures, as demonstrated for most psychiatric disorders, and thus require more time to be adequately identified. Moreover, adolescents coming from socially disadvantaged groups may encounter major barriers in accessing mental health care (63), which summed up to internal and external stigma-related issues determines reduced rates of help-seeking and possibly influences the emergence of treatment-resistance due to delay in symptom recognition. Cultural differences may also be experienced when it comes to how depression is perceived among youths belonging to different contexts and could influence crucial aspects such as the acceptance of the proposed treatments and the risk of drop-out, which significantly impacts the efficacy of treatment strategies (64).

Depressive episodes in youth populations are not of univocal interpretation and should be treated with particular attention especially in case of treatment resistance. Frequently, TRD during adolescence underpins the presence of an underlying bipolar diathesis. Indeed, most subjects who suffer from early onset bipolar disorder (BD) present a depressive polarity at their first episode, and it has been argued that up to 28% of young patients who are at first diagnosed with MDD develop subsequent hypomanic or manic episodes within 5-10 years (65). To note, bipolar depression is usually resistant to antidepressant treatment, which can also lead to a shift towards manic symptoms (66) and worsening of depression (67). Treatment strategies may differ significantly in case of a bipolar diathesis, since antidepressants should be used with caution in subjects suffering from, or at-risk for, BD, evaluating the risk/benefit ratio (68). In younger patients, bipolar depression may be difficult to distinguish from MDD, due to the depressive onset and the absence of previous episodes of opposite polarity. Some clinical features may anyway be evaluated as possible “red flags” and should thus be always considered. The main factor suggesting underlying bipolarity in youths with a depressive episode is positive familiar history for BD, which already suggests the presence of an at-risk state for BD according to previously proposed criteria (69). A positive history of psychotic disorders and suicide among first-grade relatives should also be taken into account, as well as illness characteristics, since higher severity and younger age at onset could be more frequently associated with the emergence of BD among offsprings (39). As already stated, hypomanic episodes are often under-reported or considered as ego syntonic in this population. The presence of under-threshold hypomanic symptoms should anyway be systematically investigated, and clinicians should screen patients for the presence of short periods – usually, less than four days – during which clinical features such as increased self-esteem, decreased need for sleep, talkativeness, distractibility, and increased goal-directed activity occurred (69). Course characteristics of the current depressive episode should also be considered, particularly early and abrupt onset and a positive history for recurrent depressive episodes that, as already stated, fail in responding to antidepressant treatment or present worsening of depression (39, 67). Finally, further potential predictors of underlying BD include atypical or mixed features, psychotic symptoms, psychomotor retardation, and catatonia, as well as the comorbidity with substance abuse (70–72).

When TRD occurs in early onset mood disorders, it may underpin biological, clinical, and social correlates representing possible risk factors for this condition. The first point is that depression during youth may result from altered connectivity in brain regions, e.g., amygdala, involved in emotional-affective processing and regulation, during a period when they are still under development. This can result in aberrant responses to classical antidepressant treatments, even when administered for a long period of time. Genetics plays a key role in resistance to psychopharmacotherapy, as shown by previous studies on lithium response (73, 74). The main alleles involved in TRD include those encoding for steroid hormone receptors, e.g., FKBP5 (75) and for serotonin transporter (76). Being a fast metabolizer has also been associated with a reduced response to pharmacological treatment (77). Neurometabolic alterations, such as GTP cyclohydrolase deficiency, represent additional risk factors for TRD and were thus pointed out as potentially treatable causes of this condition (78, 79). Biological sex also seems to affect response to antidepressants, since girls present higher risk of experiencing recurrence and treatment resistance (80). Neurodevelopmental disorders, such as attention deficit and hyperactivity disorder (ADHD) and autism spectrum disorders (ASD), represent additional risk factors for TRD in the young (81–84). This may be due to different reasons. First, the presence of untreated ADHD can reduce functioning, self-esteem, and treatment compliance, directly contributing to the development of depressive symptoms. In addition, the presence of ADHD is a risk factor for substance abuse, which further contributes to TRD. ADHD should thus be investigated in youths presenting with treatment-resistant mood disorders, since its clinical management can improve functioning, depressive symptoms, and treatment compliance, also reducing the risk for substance abuse. It was also reported that people with ASD are four times more likely to experience depression, especially in case of high functioning (85): however, autistic children and adolescents treated with selective serotonin reuptake inhibitors (SSRI) may have a higher risk of side effects, such as impulsive or irritable behavior and trouble sleeping (86). Other clinical factors that may contribute to the development of TRD are represented by overall greater illness severity during a depressive episode, high levels of anxiety, and suicidality (87–90). Psychiatric comorbidities, such as eating disorders and personality disorders, contribute to treatment resistance in youths who suffer from depression (36, 59). Moreover, adolescent depression is associated with a higher prevalence of substance-related disorders when compared to the general population (91), leading to increased disease severity and higher risk of TRD, especially in males (80, 92). The use of specific pharmacological agents and combinations, such as trazodone in association with fluoxetine, has been pointed out as a potential contributor to treatment resistance or symptom worsening in depressed young patients, but different confounding factors, e.g., pharmacokinetic interactions, make this findings anecdotical (93). Among comorbid medical conditions, early onset thyroid disorders may also cause depressive symptoms and contribute to TRD in the young (94). Finally, social factors including parental depression, early adversity or trauma, and belonging to a minority, have been identified as more prevalent in TRD young patients (95–99).

## 4 Treatment strategies in youth TRD

The issue of treating TRD in adolescents and young adults is challenging, due to different reasons. Indeed, no psychopharmacological treatment has specifically been approved for TRD in this population and no univocal guidelines have been reached yet. It should also be underlined that, despite some antidepressant drugs – e.g., SSRIs – are approved and considered as first-line treatments in youth depression, their use is controversial and data on their efficacy is limited. Previous literature highlighted that not only SSRIs, but also tricyclic antidepressants (TCA), present reduced effectiveness in the treatment of MDD in adolescents when compared to adult populations (100–105). Similarly, third-generation antidepressants, such as serotonin and noradrenaline reuptake inhibitors (SNRI) and mirtazapine, did not show higher efficacy when compared to placebo (101, 105). It should also be underlined that treatment indications for this age range should transcend general approaches. Indeed, there is huge variability in the clinical manifestations of depressive disorders among youths, which suggests that a precision psychiatry methodology should always be used and treatments should target symptom dimensions rather than diagnostic categories (106).

In case of non-response to the first antidepressant trial, and after considering all the factors that may impact treatment outcomes, e.g., physical and medical comorbidities (107), one possible strategy is switching to another antidepressant, usually another SSRI on a SNRI. These two treatment options showed similar response rates in previous studies (47% vs 48%, p=0.83), despite SNRIs and particularly venlafaxine presented greater effects on blood pressure and heart rates (33) In case of switching to another antidepressant, the long latency of action may represent a crucial limitation (108). As a consequence, combination and augmentation strategies are often chosen, especially in case of a partial response to the first prescribed treatment (36). As a result, adolescents with TRD frequently receive numerous psychotropic medications, including multiple drugs acting on monoaminergic systems, mood stabilizers, particularly lithium (36, 109), and atypical antipsychotics (80), but remission rates remain low and many experience adverse effects (110). The use of combination strategies in youth TRD may also present major issues in the long-term. Indeed, despite lithium appears to be a promising treatment in this population, also due to its effects in reducing self-harm and suicidality in adults (111), its clinical use in adolescents is limited by the narrow therapeutic window and by potential adverse effects, mainly those on kidney and thyroid function (36). Similarly, long-term use of second generation antipsychotics is burdened by the considerable risk of weight gain and metabolic syndrome (112), which limits their tolerability in this population.

Previous research also focused on non-pharmacological treatments, which could limit drug-related safety issues, with the strongest evidence supporting the use of cognitive behavioral therapy (CBT) in youth populations. Indeed, the efficacy of CBT has been demonstrated even in monotherapy, with evidence on its possible usefulness in relapse prevention (113, 114). As for the effectiveness of CBT as add-on treatment in TRD, it has been suggested that it should be added to psychotropic agents as early as possible, and possibly at least after one treatment failure, representing the gold standard in this population (115). On the other hand, there are also trials supporting poor response to combined CBT-antidepressant treatment, which suggests that the profile of young patients responding to psychotherapy for depressive episodes should be better characterized (116). Despite CBT being the approach with strongest literature evidence (33, 117, 118), interpersonal therapy (IPT) also demonstrated its efficacy in adolescents with TRD (119). The effectiveness of IPT for the treatment of depression arises from its focus on social and interpersonal stressors that may trigger depressive episodes and can be significantly impactful in youth populations. Due to encouraging results in adult depression, adolescent-focused IPT protocols were designed and showed to be effective (120), despite more comparative studies would be needed. Promising results were also reported for further approaches, e.g., short-term psychoanalytic psychotherapy (121), and it was thus suggested that youths failing to respond to one first trial should switch to another approach (36).

As for non-pharmacological treatments that specifically target TRD, preliminary evidence is available for physical therapies, such as transcranial magnetic stimulation (TMS), which demonstrated to be safe and tolerable in this population acting on both depressive and anxiety symptoms (122, 123). Similarly, data concerning ECT in youths is scant and its usage is limited. This may be due to restricted knowledge on the topic, caused by scantiness of clinical trials and legal restrictions in the implementation of this treatment (124, 125). Despite this, novel protocols tailored to this age group have been implemented with some positive results concerning efficacy and tolerability (90). Particularly, the use of ECT in youth populations suffering from TRD with suicidal ideation (126) or psychotic symptoms (127) appeared to be particularly effective, with response rates ranging from 50% to 90% depending on the considered report (124, 125, 128). One major issue for the use of physical therapies in adolescents and young adults could be related to possible impairment of cognitive performances, which represent one of the main domains impacting overall functioning in this population, but the tolerability profile appeared to be similar to those evidenced in adults (128).

On the basis of what stated above, the need for novel, tolerable, fast-acting antidepressants represents a crucial need in youth TRD. Several pathophysiological pathways appear to underlie TRD (129). Among the most studied mechanisms, a reduction in glutamate levels in prefrontal areas has been detected (130). This hypothesis has been supported by the antidepressant efficacy of ketamine and esketamine, modulating glutamatergic activity (131–133). At first approved as anesthetic drug in the 1970s, ketamine raised interest among the scientific community also due to its quick and long-lasting antidepressant activity (134–138). This pharmacological agent acts as a modulator of glutamatergic systems by N-methyl-D-aspartate (NMDA) antagonism (136). Other pathophysiological pathways targeted by treatment with ketamine include the modulation of prefrontal GABAergic neurons (132) and the stimulation of α-amino-3-hydroxy-5-methyl-4-isoxazolepropionic acid (AMPA) receptor (139, 140). These treatments lead to changes in neuroplasticity on mTOR/brain-derived neurotrophic factor (BDNF) signaling (141, 142). To note, the chronic use of ketamine is also associated with increase in blood neurotrophins, such as BDNF (143).

Despite clinical recommendations for TRD have been developed during the last decades (see, e.g (144), widely accepted guidelines have not been implemented yet. Anyway, increasing evidence is being provided for what concerns recently approved treatments for TRD, among which the main one is intranasal esketamine (145). Emerging evidence supports the use of ketamine and esketamine in youths with TRD. As already elucidated, several randomized studies demonstrated that ketamine infusion lead to significant reduction in depressive symptoms compared to placebo in TRD (138, 146–149). Recent evidence also showed that ketamine may act on suicidal ideation with rapid action and minimal side effects (131, 150–157). Since the use of SSRI in adolescents suffering from depression has been associated with increased suicidal risk (158), with a pooled relative risk of 1.28 (95% CI: 1.09–1.51) as detected by a recent meta-analytic study (159), the possible effect of ketamine on this dimension gains even higher importance. Another psychopathological domain on which antidepressants were demonstrated to exert low effectiveness in young populations is anhedonia (87), possibly representing another promising target of treatment with ketamine as demonstrated by previous reports on bipolar depression, demonstrating reductions of anhedonia levels at different times during the 14 days after the infusion, not depending on the effect on other depressive dimensions (160). Despite literature on the topic is still scant, recent studies showed encouraging results concerning the use of intravenous ketamine in youth populations. Indeed, a randomized controlled trial pointed out towards the efficacy of one single dose of intravenous ketamine, compared to active placebo (midazolam), in reducing depressive symptoms in adolescents who did not respond to previous treatments (161). During the 3 days following infusion, the prevalence of response to ketamine treatment was 76% (p=0.046) compared with 35% of responders in the active placebo group, with a mean difference in the Montgomery-Åsberg Depression Rating Scale (MADRS) of -8.69 ± 15.08 and an effect size of 0.78 (161, 162). Further data from open-label trials and case reports/case series also suggested that low-dose ketamine had a rapidly acting antidepressant effect in adolescents. In particular, an open label trial investigating the efficacy of six ketamine infusions (0.5 mg/kg) over two weeks on adolescent (12-18 years old) TRD demonstrated a reduction of 42.5% (p.<0.01) at the Children’s Depression Rating Scale - Revised (CDRS-R) (163, 164). Intravenous ketamine treatment appeared to be safe and well-tolerated in this population, with transitory dissociative and hemodynamic symptoms that resolved after few hours (161, 163, 165). The neural correlates of intravenous ketamine treatment in adolescent TRD may reside in the reduced activation of corticolimbic, corticostriatal, and default mode networks, which underpins an increase in hedonic capacity associated with reduced negativity bias and attitude towards positivity (166). To note, the use of ketamine would appear more appropriate among specific sub-populations of TRD patients, as confirmed by adult studies. Indeed, it has been suggested that the most appropriate use of ketamine would be as a short-term treatment in acute settings, especially in case of suicidal risk, which appears to decrease even before the improvements of depressive symptoms (167). Anyway, the latter were faster than those obtained with other antidepressant drugs, e.g., SSRIs (168). Moreover, studies conducted in adult populations elucidated that efficacy on specific domains, e.g., cognitive symptoms, was obtained only in TRD patients with anxious depression when compared to non-anxious ones (164). Similarly, it can be hypothesized that response to ketamine treatment in youths could depend on specific factors. Most evidence so far showed that response to ketamine in youth populations is associated with a shorter duration and lower severity of the current episode, treatment with SSRIs rather than SNRIs, and ADHD comorbidity (169).

Esketamine, the S-enantiomer of ketamine, was demonstrated to be a precious therapeutic option when combined with serotoninergic drugs in adult TRD (170) and was thus approved by regulatory agencies for the treatment of this condition (171–173). Due to its higher affinity for NMDA receptor and to its intranasal formulation, esketamine offers interesting therapeutic perspectives for outpatient use, and is thus the only treatment approved for TRD in European countries when combined with SSRIs or SNRIs (174). Previous reports showed promising results concerning the use of esketamine in adolescent populations. In a randomized-controlled trial, three intravenous infusions of esketamine (0.25 mg/kg) were associated with higher antidepressant and anti-suicidal effects according to greater reductions in the scoring of depression severity (MADRS total score mean changes: -15.3 ± 11.2 vs -8.8 ± 9.4, p=0.002) and suicide-related measures (Columbia Suicide Severity Rating Scale (CSSRS) ideation score mean changes: -2.6 ± 2 vs -1.7 ± 2.2, p=0.007; CSSRS intensity score mean changes: -10.6 ± 8.4 vs -5 ± 7.4, p=0.002) when compared to active placebo in adolescents aged 13-18 (175). In the esketamine treatment group, a significant improvement in some cognitive domains, particularly processing speed (drug main effect: F=6.607, p=0.013) was also observed, while no impairment of the other domains were reported (176). A double-blind, randomized, midazolam-controlled study of intranasal esketamine for adolescents with MDD at imminent risk of suicide showed that pooled esketamine doses (56 mg, 84 mg) were superior over midazolam in reducing CDRS-R at 24 hours after the initial dose (p=0.037), with relatively low incidence of serious adverse events (28 mg: 13.8%, 56 mg: 22.6%, 84 mg: 4.3%) that did not cause treatment discontinuation in any case (177). Further evidence coming from case report studies (178) confirmed that intranasal esketamine use in youth populations could be tolerable with minimum adverse reactions, despite its efficacy has not been proved yet (175, 176). As for possible issues related to the use of ketamine and esketamine in the long-term, it has been argued that vigilance for possible cognitive effects and the emergence of abuse is essential, despite preliminary data on adult population is encouraging (179). Current evidence for intravenous ketamine treatment in adolescent populations is based on preclinical studies. Despite most of these showed no later cognitive impairment and an overall good tolerability in the long-term with the administration of low-dose ketamine (180, 181), further data underlines a reduction of spatial working memory together with morphological and degenerative brain changes when administering higher doses to adolescent rats (182). This suggests that detrimental effects of ketamine on brain development may depend on the chronicity and dose of administration, but it should also be noted that clinical doses of this medication are significantly lower than those used in these experimental settings (183). As for esketamine, no long-term studies assessed its safety in adolescent populations. To note, encouraging results come from trials conducted in clinical settings evaluating long-term esketamine use in adults, which showed overall good tolerability, with no relevant incidence of serious adverse effects, including abuse (173, 184, 185).

To note, further treatments for TRD are being evaluated, such as psychedelics and cannabidiol, with promising results in adult populations but no preliminary evidence, including preclinical studies, in adolescents (108). Among classical psychedelics, psylocibin is being evaluated for the treatment of affective - and particularly depressive - disorders, with preliminary clinical evidence in adult populations (186). Based on animal models, the antidepressant efficacy of this compound may rely on its effect on 5HT-2A receptors, possibly increasing serotonin and glutamate levels and modulating the excitability of pyramidal neurons and synaptic plasticity processes (187). As for cannabidiol, its possible usefulness in the improvement of affective and stress-induced symptoms has been widely proved by preclinical studies, despite its efficacy may vary on the basis of different biological determinants, e.g., sex (188, 189). Preclinical evidence is also available for the use of the compound in adolescence, confirming its effectiveness at lower doses than those needed in adulthood (190). Contrasting results on cannabidiol length of action in adolescent rodents were provided, suggesting that the fast-acting antidepressant action may not be sustained over time (190, 191), but these data require further validation. Since evidence for novel antidepressant strategies has not been confirmed by clinical studies on adolescent populations and preclinical data is still scant, no conclusions can be drawn on possible uses of these molecules in youths so far.

Despite some novel agents, particularly ketamine and esketamine, have demonstrated to be rapid and effective treatments, it is crucial to correctly assess the risk-benefit ratio for each subject, to make informed decisions about treatment appropriateness, especially in vulnerable populations. Indeed, together with the already-cited dissociative symptoms, further side effects, such as alterations in blood pressure levels and the risk of developing addiction, should be considered. Moreover, not all subjects with TRD respond positively to this treatment: severity of depression, comorbidities, and previous treatment history should be considered in order to select individuals that are most likely to benefit from the treatment (192). Is should also be noticed that the long-term impact of these drugs on young people’s physical, cognitive, and emotional development has not been fully studied yet (193). Further research is thus needed in order to consider these aspects, together with legal and ethical issues that safeguard the well-being of young subjects with TRD (194). Providing psychiatric care to young people, and particularly minors, rises a series of ethical and legal concerns that fully reflect the dynamism of this field, always facing new challenges related to broader changes at societal level. One major issue is the capacity of providing informed consent to treatments, which remains controversial. In case of minors, parents have the power to guide treatment choices, expressing their preference and eventually outweighing children’s willingness to undergo specific therapies (195). On the other hand, there are specific jurisdictions that consider the possibility of adolescents with a clear understanding of their condition expressing their informed consent, as well as emancipation following marriage or economic independence (196). Prescribing principles are similar for adult and adolescent populations and are based on beneficence and nonmaleficence, since clinicians should always evaluate the best treatment choice for their patients and avoid short- and long-term harmful effects (197). To note, most prescriptions in adolescent populations may come from psychiatrists working with adults and not receiving specific training. In this context, prescription of specific pharmacological agents, e.g., antidepressants, may result in further criticalities, being burdened by warnings due to possible adverse effects and issues like worsening of depression and the emergence of suicidal ideation. This may be particularly relevant in case of new pharmacological strategies that have not been studied in depth in youth, and particularly adolescent, populations, due to scant knowledge concerning their possible effects on rapidly developing brains. Apart from the already-elucidated possible effects on cognitive function and the development of structural and functional alterations in the central nervous system (198), it should also be noted that depressive symptoms in youths could underpin the later emergence of further conditions, such as schizophrenia-spectrum disorders (178). In this case, the use of ketamine and esketamine should be cautious due to the potential influence on the development of psychosis (199).

Several challenges need to be addressed to ensure adequate treatment for youth populations suffering from TRD, as well as appropriate and safe usage of new pharmacological agents in these patients. Indeed, current literature on the topic of youth TRD lacks a precision approach in the identification of this major health problem, since a univocal definition has not yet been provided and symptom domains that could more clearly characterize the clinical picture have not been examined in depth. Addressing these issues would help a more accurate selection of patients included in clinical trials, helping to establish treatment efficacy and safety of novel pharmacological agents. Studies on fast-acting antidepressants in youths with TRD are scant and are based on small sample sizes, not always following a randomized-controlled design, which limits the generalizability of their findings. This is also a major issue that contributed to the narrative approach of this review, since the possibility of a quantitative and meta-analytic synthesis of the study results was lacking. Despite this, we believe that the promising results obtained by preclinical and clinical studies warrant further investigation. Moreover, we should underline that differential diagnosis, risk assessment, patient selection, long-term monitoring, and ethical concerns are crucial elements to consider even since the diagnostic process and should contribute to treatment choices in youth TRD. A multidisciplinary approach is important for optimizing the usage of ketamine and esketamine in youth populations, and safeguard the well-being of subjects during and after the treatment process, also considering that integrated treatments are expected to be proposed since very early illness stages in order to reduce the overall impact of youth psychopathology on overall well-being (200, 201).

## 5 Conclusions

Resistance to treatment during a major depressive episode remains a relevant challenge in youth populations, with different possible correlates including an unrecognized bipolar diathesis. The lack a of a univocal definition of this condition suggests that future research on the topic is needed in order to better clarify its clinical correlates. Further characterization of this condition is strongly needed also in order to optimize treatment strategies in a precision psychiatric perspective that goes beyond traditional nosographic descriptions. Moreover, treatments suggested for youth treatment-resistant depression are not population-specific and the current clinical practice advocates the use of strategies that follow those adopted in adults. Despite this, the use of traditional antidepressant agents in adolescents and young adults is burdened by effectiveness and safety concerns. Further research on integrated treatment strategies should clarify the role of non-pharmacological interventions in this population, also considering new psychotherapeutic approaches and psychosocial treatments. Results from this narrative review also suggest that fast-acting antidepressants are promising in this population and appear to be well-tolerated and effective. Despite this, the scantness of clinical studies, the limitations posed by ethical and legal issues, together with the lack of long-term safety and effectiveness data, advises that further research on the topic would be needed. Future studies are thus expected to provide further evidence concerning this population, where risk-benefit ratio should always be taken into account when addressing treatment choices.

## Author contributions

GM: Conceptualization, Writing – review & editing. GCi: Writing – original draft. FS: Writing – original draft. MC: Writing – original draft. GCa: Writing – original draft. PB: Writing – review & editing. FDG: Writing – review & editing. PM: Writing – review & editing. AT: Supervision, Writing – review & editing.
